# The prognostic value of different HPV infection statuses in cervical cancer

**DOI:** 10.1038/s41598-026-58010-2

**Published:** 2026-06-14

**Authors:** Yu Sun, Ping Liu, Mei Ji, Min Hao, Qinlan Luo, Zhumei Cui, Xiaoyan Xin, Donglin Li, Mingwei Li, Weili Li, Chunlin Chen

**Affiliations:** 1 Department of Obstetrics and Gynecology, Nanfang Hospital, Southern Medical University, Guangzhou, China; 2 Department of Obstetrics and Gynecology, Dalian Women and Children’s Medical Group, Dalian, China; 3 Department of Obstetrics and Gynecology, First Affiliated Hospital of Zhengzhou University, Zhengzhou, China; 4 Department of Gynecology, The Second Hospital of Shanxi Medical University, Taiyuan, China; 5 Department of Obstetrics and Gynecology, The Affiliated Hospital of Qingdao University, Qingdao, China; 6 Department of Gynecology, Xijing Hospital, Xijing, China; 7 Department of Obstetrics and Gynecology, Guizhou Provincial People’s Hospital, Guizhou, China; 8 Department of Gynecology, Jiangmen Central Hospital, Jiangmen, China

**Keywords:** HPV negative, Cervical cancer, Radical surgery, Prognosis, Real-world study, Cancer, Diseases, Oncology

## Abstract

To compare oncological outcomes between HPV-positive and HPV-negative cervical cancer patients in a large Chinese multicenter cohort. This retrospective study included cervical squamous cell carcinoma, adenocarcinoma, and adenosquamous carcinoma. Propensity score matching (PSM, 2:1) was used to balance baseline characteristics. Kaplan–Meier method and Cox proportional hazards regression were applied to compare 5-year overall survival (OS) and disease-free survival (DFS). The proportional hazards assumption was tested. Multicenter heterogeneity was adjusted using center stratification. Missing data were managed by complete-case analysis and multiple imputation sensitivity analysis. A total of 11,215 eligible patients were included. Before PSM, the HPV-positive group showed significantly better 5-year OS (92.3% vs. 84.3%, *P* < 0.001) and DFS (88.0% vs. 79.6%, *P* < 0.001) than the HPV-negative group. Multivariable Cox analysis confirmed HPV negativity as an independent risk factor for OS (HR = 1.545, 95%CI 1.091–2.188, *P* = 0.014) and DFS (HR = 1.556, 95%CI 1.212–1.997, *P* < 0.001). After 2:1PSM, HPV-positive patients still had superior 5-year OS (88.4% vs. 84.3%, *P* = 0.004) and DFS (85.3% vs. 79.6%, *P* < 0.001). Among 7,998 patients who underwent radical surgery, similar results were observed before and after PSM. HPV-negative status is associated with inferior 5-year OS and DFS and serves as an independent adverse prognostic factor in cervical cancer, including patients treated with radical surgery.

**Supplementary Information:**

The online version contains supplementary material available at https://doi.org/10.1038/s41598-026-58010-2.

## Introduction

Cervical cancer (CC) is the highest incidence of malignant tumors affecting the female reproductive system, posing a serious threat to women’s health and life^1^. Human papillomavirus (HPV) has been confirmed as the primary pathogen of cervical cancer. Persistent infection with High-risk human papillomavirus (HR-HPV) is the primary cause of cervical intraepithelial neoplasia (CIN) and cervical cancer^2^, With the widespread implementation of HPV-based screening programs, more preinvasive lesions are detected early; however, some patients experience delayed diagnosis due to false-negative results or limited access to screening.

Cervical cancer shows distinct histological distributions: squamous cell carcinoma accounts for more than 80% of cases, while adenocarcinoma, adenosquamous carcinoma, and other rare subtypes are less common. Non-squamous histologies are associated with worse prognosis, lower treatment sensitivity, higher rates of HPV negativity, and infection with non-classic HR-HPV genotypes^3^.

Most studies focus on HPV-positive cervical cancer, while HPV-negative cervical cancer remains underinvestigated. Previous reports on the prognostic impact of HPV status are inconsistent. Some studies indicate that HPV-negative cervical cancer is more often diagnosed at advanced stages and carries a poor prognosis^4^. Other studies suggest no significant prognostic correlation^5^. HPV-negative cervical cancer may represent a distinct biological subtype with unique molecular features, including different signaling pathway mutation profiles^6^. However, due to its low incidence and limited research, the underlying mechanisms remain unclear^4^.

This large multicenter retrospective study aimed to evaluate the prognostic value of HPV status in cervical cancer, with a focus on survival outcomes before and after propensity score matching, as well as in patients treated with radical surgery.

## Materials and methods

### Study design and data source

This multicenter retrospective cohort study was approved by the Ethics Committee of Nanfang Hospital, Southern Medical University (approval number NFEC-2017–135; clinical registration number ChiCTR1800017778). The Four C database was established with 47 hospitals in mainland China and included 63,926 cervical cancer patients diagnosed between 2004 and 2018. Data collection, double-entry verification, and follow-up procedures have been described previously^7^. Clinical stage was updated according to the 2018 FIGO staging system^8^.

### Inclusion and exclusion criteria

Inclusion criteria: 1.Age ≥ 18 years 2.Histologically confirmed cervical squamous cell carcinoma, adenocarcinoma, or adenosquamous carcinoma 3.Available HPV test results (positive or negative) 4.Complete survival and follow-up data.

For the surgical subgroup: 1.Initial treatment with radical surgery 2.Type QM-B or QM-C hysterectomy plus pelvic lymphadenectomy ± para-aortic lymphadenectomy.

Exclusion criteria: 1.Special clinical conditions: pregnancy-associated cervical cancer, incidental occult cervical cancer, cervical stump carcinoma, and synchronous malignancy of other organs.2.Missing key survival or covariate data (handled by sensitivity analysis).

### HPV testing

All patients were tested for HPV using in-house real-time polymerase chain reaction (PCR) targeting 13 high-risk HPV genotypes. For patients with an initial negative HR-HPV result, a second sampling and repeat testing were performed using the same method. Only patients with two consecutive negative results were defined as HPV-negative. All 47 centers adopted the same HPV detection assay, with minor variations in experimental procedures across individual centers.However, potential heterogeneity in testing conditions during 2004–2018 cannot be fully excluded.

### Outcome measures

The primary endpoints were 5-year overall survival (OS) and 5-year disease-free survival (DFS).

OS: time from diagnosis to death from any cause or last follow-up.

DFS: time from diagnosis to disease recurrence, cancer-related death, or last follow-up.

### Statistical methods

Continuous data were presented as mean ± standard deviation; categorical variables were summarized as percentages. Between-group differences were analyzed using the independent t-test or χ^2^ test. Survival curves were constructed using the Kaplan–Meier method and compared by log-rank test. The proportional hazards assumption was verified using Schoenfeld residuals. Center-level heterogeneity was adjusted by center stratification in all Cox models. Missing data: Missing rates for each covariate are shown in Tables 1 and 2 (e.g., 51.3% missing for tumor diameter). Complete—case analysis was used as the primary method, and sensitivity analysis was used to validate robustness.Selection bias due to missing data was acknowledged as a limitation.Propensity score matching (PSM): 2:1matching was performed with a caliper width of 0.2. Variables included in the PSM model were: age, FIGO stage, pathological type, tumor diameter, Depth of cervical stromal invasion, LVSI, parametrial invasion, vaginal margin status, and abdominal pelvic lymph node metastasis. The score was estimated using logistic regression. After PSM, multivariate Cox regression was repeated. We adopted the multiple imputation by chained equations (MICE) to handle missing data for sensitivity analysis. The imputation model included the following covariates: age, FIGO stage, pathological type, tumor diameter, depth of cervical stromal invasion, lymphovascular space invasion, parametrial invasion, vaginal margin status, and abdominopelvic lymph node metastasis. All analyses used SPSS 22.0. *P* < 0.05 was considered statistically significant.

**Table 1:** Comparison of baseline data before and after matching between HPV-positive and HPV-negative cervical cancer patients.

Parameter	Before matching					After matching(PSM,2:1)				
	HPV-positive group (n = 10,027)		HPV-negative group (n = 1188)		*P*-value	HPV-positive group (n = 2376)		HPV-negative group (n = 1188)		*P*-value
Age	47.79 ± 9.89		48.44 ± 9.42		< 0.001	47.32 ± 9.06		47.08 ± 9.20		0.479
FIGO staging					< 0.001					0.941
I	5122	51.0%	497	41.8%		1297	54.6%	497	41.8%	
II	2209	22.0%	323	27.2%		457	19.2%	323	27.2%	
III	1771	17.6%	240	20.2%		386	16.3%	240	20.2%	
IV	40	0.4%	13	1.1%		51	2.1%	13	1.1%	
Cancer staging is not detailed	885	8.8%	115	9.7%		185	7.8%	115	9.7%	
Pathological type					< 0.001					0.523
Squamous cell carcinoma	9115	90.9%	957	80.6%		2096	88.2%	957	80.6%	
Adenocarcinoma	744	7.4%	206	17.3%		226	9.5%	206	17.3%	
Adenosquamous Carcinoma	168	1.7%	25	2.1%		54	2.3%	25	2.1%	
Tumor diameter					0.187					0.433
≤ 4 cm	4123	41.1%	445	37.4%		1056	44.4%	445	37.5%	
> 4 cm	760	7.6%	96	8.1%		160	6.8%	96	8.9%	
Unknown	5144	51.3%	647	54.5%		1160	48.8%	647	54.5%	
Depth of cervical stromal invasion					0.002					0.884
≤ 1/2 muscle layer	3851	38.4%	393	33.1%		950	40.0%	393	33.1%	
> 1/2 muscle layer	3359	33.5%	424	35.7%		806	33.9%	424	35.7%	
Unreported	2817	28.1%	371	31.2%		620	26.1%	371	31.2%	
LVSI					0.001					0.858
Negative	6587	65.7%	779	65.6%		1599	67.3%	779	65.6%	
Positive	1535	15.3%	126	10.6%		286	12.0%	126	10.6%	
Unreported	1905	19.0%	283	23.8%		491	20.7	283	23.8%	
Parametrial invasion					0.718					0.205
Negative	8609	85.9%	957	80.6%		2003	84.3%	957	80.6%	
Positive	130	1.3%	13	1.1%		28	1.2%	13	1.1%	
Unreported	1288	12.8%	218	18.4%		345	14..5	218	18.4%	
Vaginal margin					0.093					0.553
Negative	8152	81.3%	920	74.4%		1896	80.1%	920	74.4%	
Positive	168	1.7%	18	1.5%		32	1.0%	18	1.5%	
Unreported	1707	17%	250	21.1%		448	18.9%	250	21.1%	
Abdominal pelvic lymph node metastasis					0.806					0.471
None	6412	64.0%	736	62.0%		1526	64.2%	736	62.0%	
Exist	1294	12.9%	152	12.8%		285	12.0%	152	12.8%	
Unreported	2321	23.1%	300	25.2%		565	23.8%	300	25.2%

**Table 2:** Cox analysis of HPV-positivity group and HPV-negative group for cervical cancer under the inclusive criterion.

Factor	*P*-value	HR	95% CI	
			LOWER	UPPER
*Before matching*				
OS				
Age	0.045	1.013	1.000	1.027
FIGO staging	< 0.001	1.021	1.002	1.040
HPV status	0.014	1.545	1.091	2.188
Depth of cervical stromal invasion	< 0.001	1.354	1.027	1.672
LVSI	0.001	2.239	1.888	2.644
DFS				
HPV status	0.001	1.432	1.212	1.997
FIGO staging	< 0.001	2.224	1.593	2.771
Depth of cervical stromal invasion	< 0.001	1.336	1.208	2.272
LVSI	0.007	1.441	1.013	2.765
Abdominal pelvic lymph node metastasis	0.021	4.462	3.253	6.253
*After matching*				
OS				
HPV status	0.011	1.556	1.081	2.008
FIGO staging	0.001	1.323	1.184	1.652
Depth of cervical stromal invasion	< 0.001	1.336	1.208	2.272
DFS				
HPV status	0.001	1.376	1.201	1.848
FIGO staging	0.002	1.274	1.096	1.480
Depth of cervical stromal invasion	0.013	1.240	1.047	1.469
Abdominal pelvic lymph node metastasis	< 0.001	3.628	2.912	4.522

Before PSM, covariate sets for OS and DFS were configured according to clinical prognostic relevance with minor discrepancy in individual variables. After PSM, LVSI and other covariates were omitted in multivariate Cox model owing to post-matching imbalance and low statistical contribution, and only clinically critical and well-balanced variables were retained.

## Result

### Survival outcomes in all eligible HPV-positive and HPV-negative cervical cancer patients

#### Patient enrollment

**Fig. 1:**
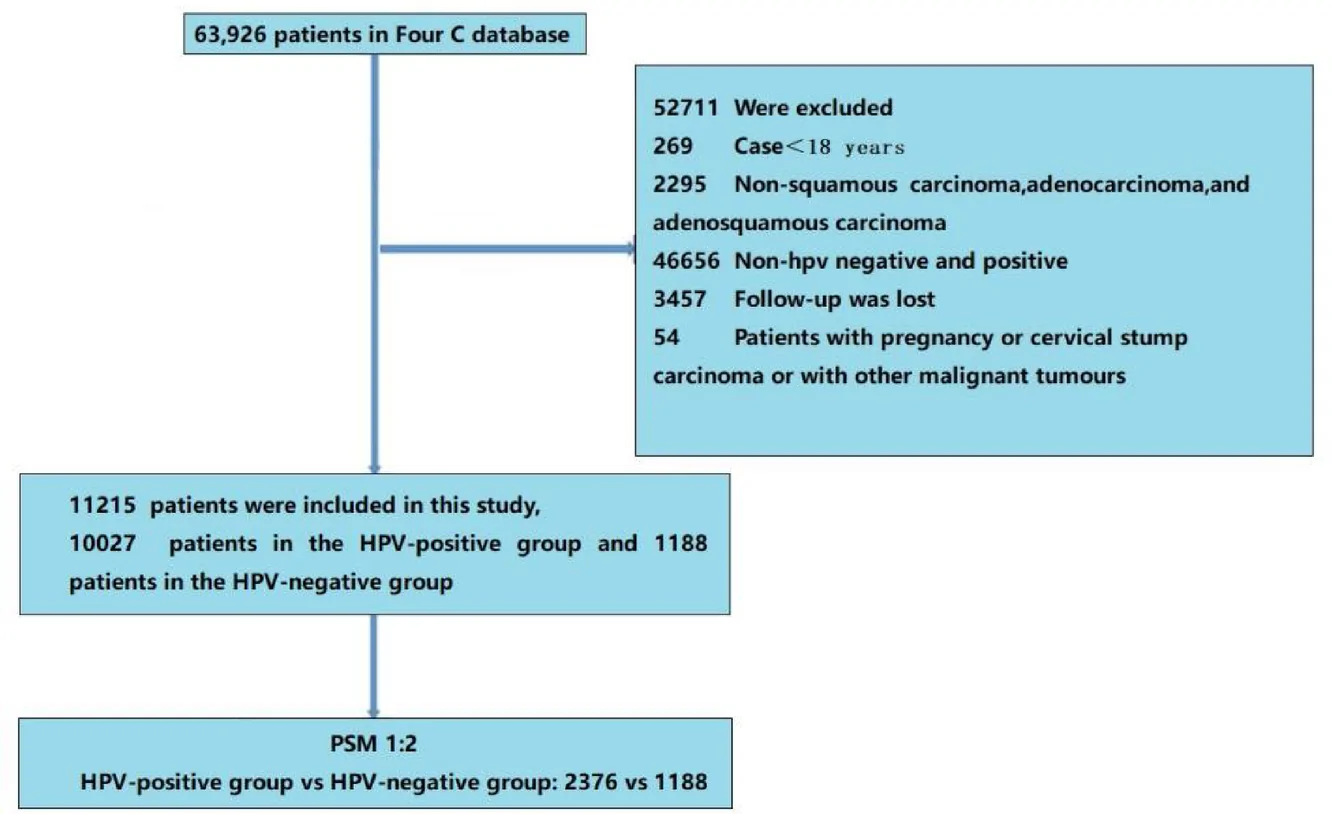
Comparison of oncological outcomes for HPV-positive and HPV-negative cervical cancer screening process.

A total of 11,215 patients were included after screening: 10,027 HPV-positive and 1,188 HPV-negative. Median follow-up was 49 months; 595 deaths (5.3%) and 1,051 recurrence/death events (9.4%) were observed (Fig. 1).

#### Before matching

In total, 11,215 patients with cervical cancer were stratified into 10,027 HPV-positive and 1,188 HPV-negative cases. Significant differences were noted between the two groups in age, FIGO stage, histological type, Depth of cervical stromal invasion, and lymphovascular space invasion (LVSI). HPV-negative patients were older, with more advanced FIGO stage, deeper cervical stromal invasion, higher LVSI positivity, and a significantly higher proportion of adenocarcinoma/adenosquamous carcinoma (19.4% vs 9.5%, *P* < 0.001) (Table 1).

**Fig. 2:**
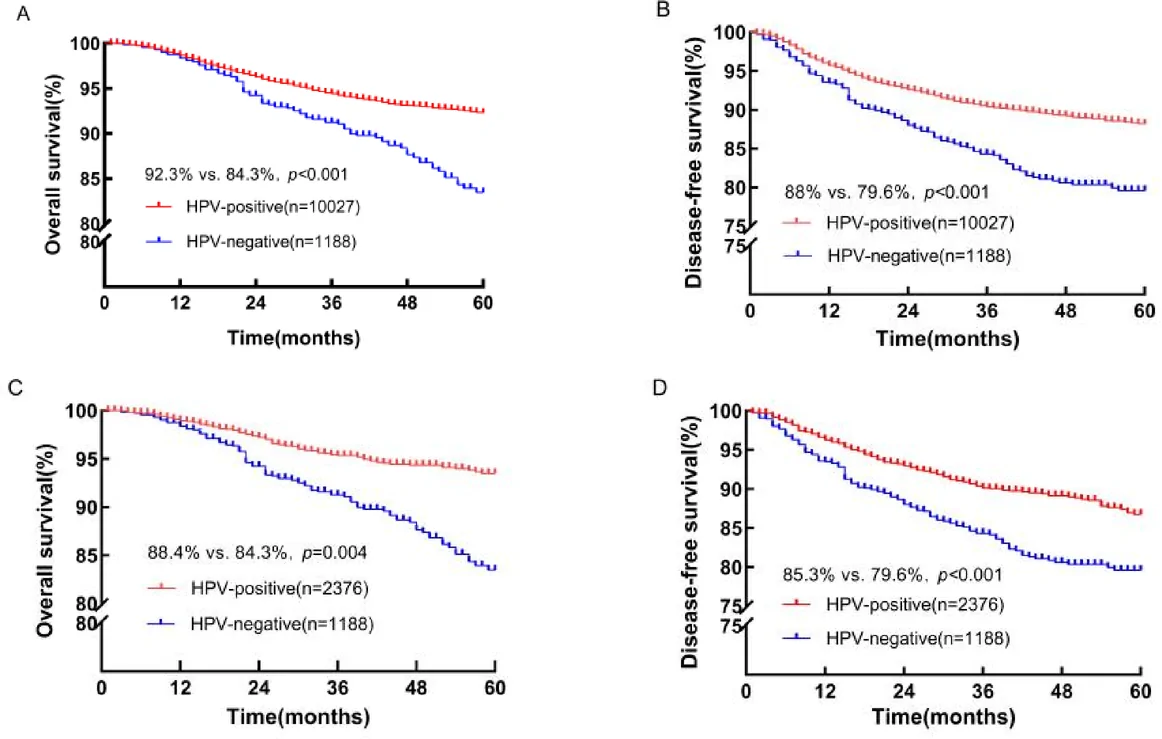
Survival curves of cervical cancer patients in HPV-positive and HPV-negative groups(unadjusted, panels (**A**, **B**); adjusted, panels (**C**, **D**).

The HPV-negative group showed higher 5-year mortality and recurrence rates, with significantly poorer 5-year OS and DFS than the HPV-positive group (both *P* < 0.001) (Fig. 2).

Multivariate Cox analysis showed that HPV negativity was independently associated with poorer OS (*P* = 0.014) and DFS (*P* < 0.001) (Table 2).

#### After 2:1PSM

After PSM, baseline variables were well balanced between 2,376 HPV-positive and 1,188 HPV-negative patients (all *P* > 0.05) (Table 2).

At 5 years, 109 deaths (4.6%) occurred in the HPV-positive group compared with 106 deaths (8.9%) in the HPV-negative group; the 5-year OS rates were 88.4% versus 84.3% (*P* = 0.004). There were 204 recurrence/death events (8.6%) in the HPV-positive cohort and 179 events (15.1%) in the HPV-negative cohort, with 5-year DFS rates of 85.3% and 79.6%, respectively (*P* < 0.001), as shown in Fig. 2. HPV negativity remained an independent risk factor for OS (*P* = 0.011) and DFS (*P* < 0.001)(Table 2).Sensitivity analysis using multiple imputation produced consistent findings. HPV-negative status remained an independent adverse prognostic factor for both OS and DFS, with HR and 95% CI closely matching those from the complete-case analysis, which confirms the robustness of our primary results (Table S1).

### Survival outcomes in patients undergoing radical surgery

#### Patient enrollment

**Fig. 3:**
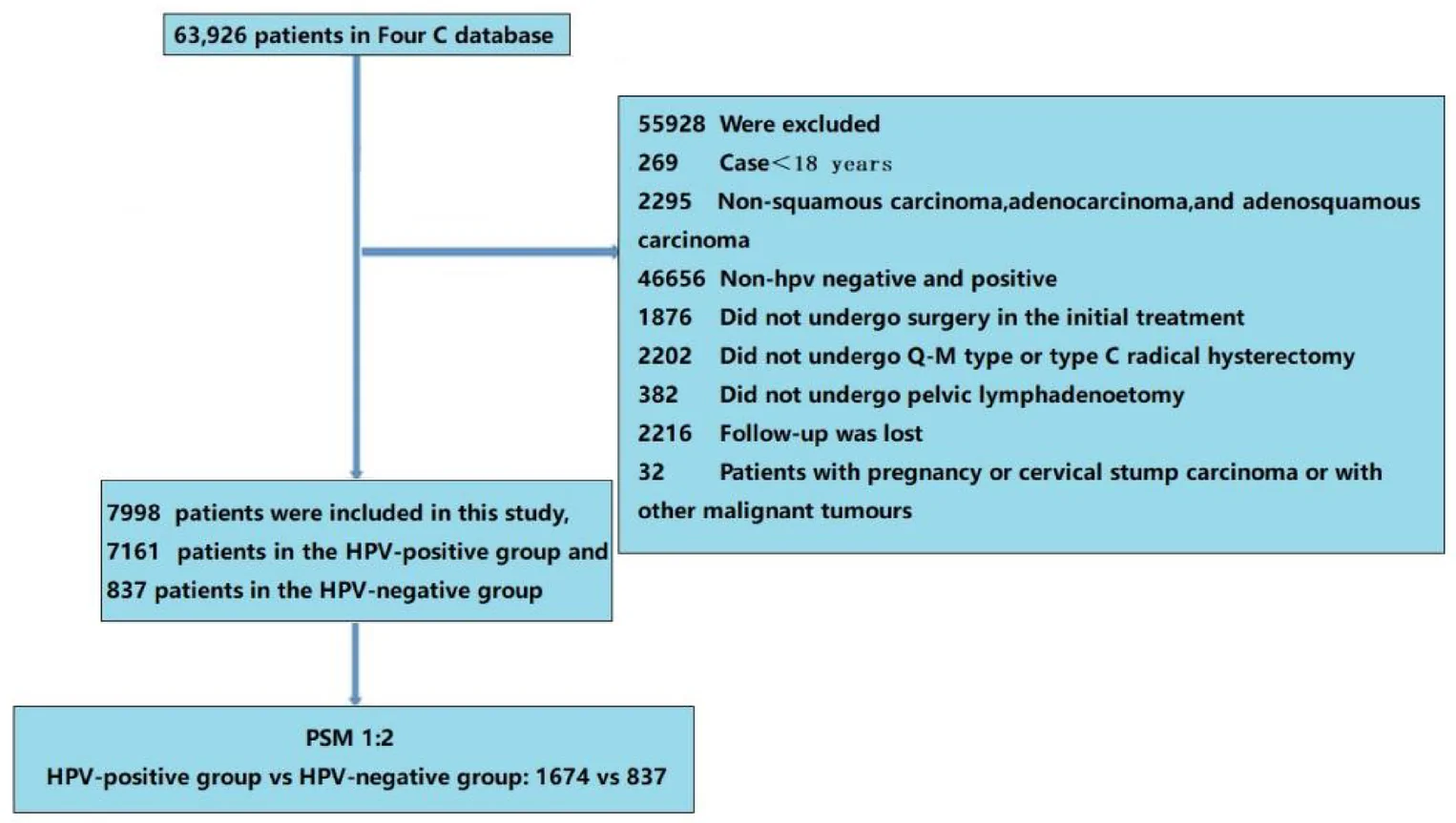
Screening process for oncological outcomes after radical surgery for HPV-positive and HPV -negative cervical cancer.

After inclusion and exclusion screening, 7,998 patients were enrolled with a median follow-up of 59 months. There were 324 deaths (4.1%) and 680 recurrence/death events (8.5%). Patients were stratified into 7,161 HPV-positive and 837 HPV-negative cases (Fig. 3).

#### Before matching

Oncological outcomes were analyzed in 7,998 cervical cancer patients (7,161 HPV-positive, 837 HPV-negative). Baseline FIGO stage, pathological type and LVSI differed significantly; HPV-negative patients had higher LVSI positivity and more adenocarcinoma/adenosquamous carcinoma (19.1% vs. 9.3%, *P* < 0.001) (Table 3).

**Table 3:** Comparison of baseline data before and after matching between HPV-positive and HPV-negative cervical cancer radical surgery patients.

Parameter	Before matching					After matching(PSM,2:1)				
	HPV positive group(n = 7161)		HPV negative group(n = 837)		*P*-value	HPV positive group(n = 1674)		HPV negative group(n = 837)		P-value
Age	47.39 ± 9.32		47.28 ± 8.77		0.745	47.32 ± 9.06		47.08 ± 9.20		0.479
FIGO staging					0.001					0.941
I	4063	56.70%	415	49.70%		977	58.40%	415	49.70%	
II	1685	23.50%	244	29.20%		384	22.90%	244	29.20%	
III	1226	17.10%	144	17.20%		278	16.60%	144	17.20%	
IV	3	0.0%	0	0.0%		0	0	0	0.0%	
Cancer staging unknown	184	2.6%	34	4.1%		35	2.1%	34	4.1%	
Pathological type					< 0.001					0.413
Squamous cell carcinoma	6373	89%	651	77.8%		1498	89.5%	651	77.8%	
Adenocarcinoma	642	9.0%	164	19.6%		145	8.7%	164	19.6%	
Adenosquamous carcinoma	146	2.0%	22	2.6%		31	1.9%	22	2.6%	
Tumor diameter					0.262					0.433
≤ 4 cm	3735	52.2%	404	48.3%		866	51.8%	404	48.3%	
> 4 cm	716	10%	89	10.6%		180	11%	89	10.6%	
Unknown	2710	37.8%	344	41.1%		628	37.5%	344	41.1%	
Depth of cervical stromal invasion					0.112					0.884
≤ 1/2 muscle layer	3092	43.2%	338	40.4%		726	43.4%	338	40.4%	
> 1/2 muscle layer	3176	44.4%	403	48.1%		786	47.0%	403	48.1%	
Unreported	893	12.5%	96	11.5%		162	9.7%	96	11.5%	
LVSI					< 0.001					0.858
Negative	5289	73.9%	673	80.4%		1232	73.6%	673	80.4%	
Positive	1453	20.3%	116	13.9%		326	19.5%	116	13.9%	
Unreported	419	5.8%	48	5.7%		116	6.9%	48	5.7%	
Parametrial invasion					0.546					0.205
Negative	7038	98.3%	825	98.6%		1646	98.3%	825	98.6%	
Positive	123	1.7%	12	1.4%		28	1.7%	12	1.4%	
Vaginal margin					0.141					0.553
Negative	6846	95.6%	810	96.8%		1586	94.7%	810	96.8%	
Positive	156	2.2%	17	2%		38	2.3%	17	2%	
Unreported	159	2.2%	10	1.2%		50	3%	10	1.2%	
Abdominal Pelvic Lymph Node Metastasis					0.919					0.471
None	5922	82.7%	691	82.6%		1366	81.6%	691	82.6%	
Exist	1239	17.3%	146	17.4%		308	18.4%	146	17.4%	

**Fig. 4:**
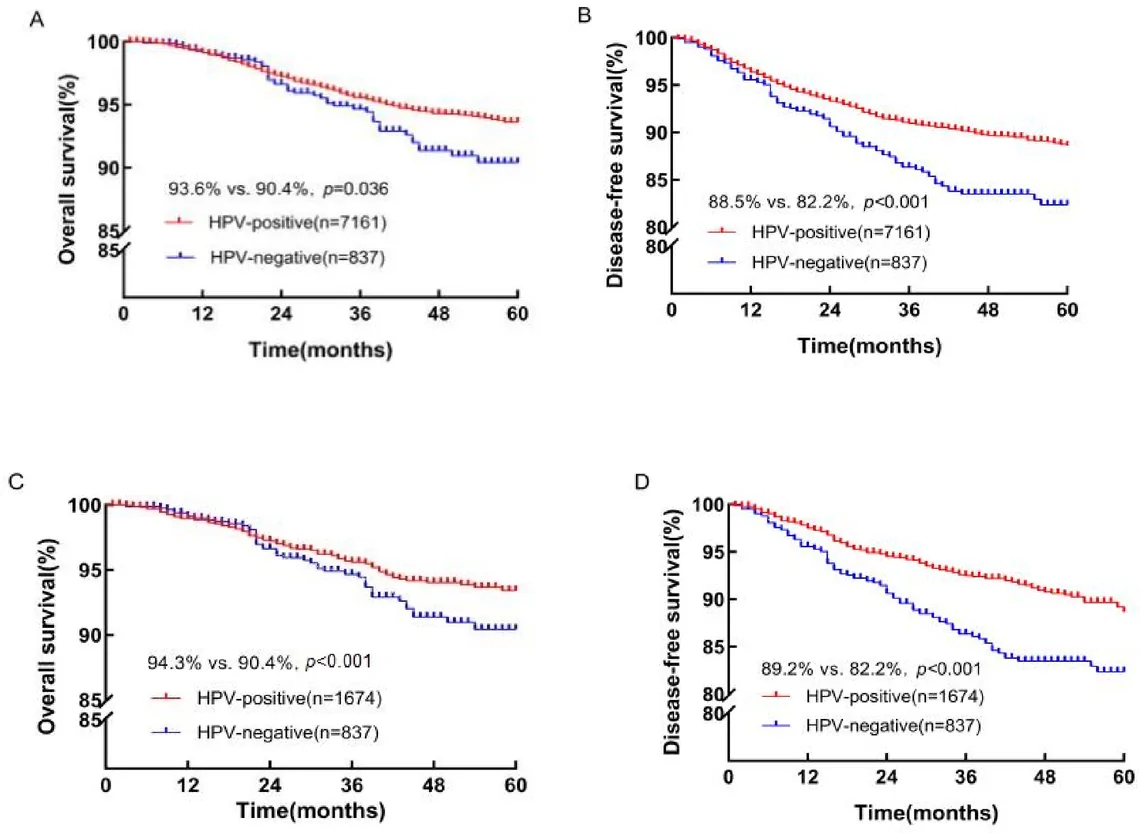
Survival curves of patients after radical cervical cancer surgery in HPV-positive group and HPV-negative group. (unadjusted, panels (**A**, **B**); adjusted, panels (**C**, **D**).

HPV-positive patients had lower 5-year mortality (3.9% vs. 5.6%), better 5-year OS (93.6% vs. 90.4%, *P* = 0.036), fewer recurrence/death events (8.0% vs. 12.8%) and superior 5-year DFS (88.5% vs. 82.2%, *P* < 0.001) (Fig. 4).

Cox regression identified HPV status, FIGO stage, Depth of cervical stromal invasion and LVSI as independent prognostic factors for mortality (*P* = 0.041) and HPV status, invasion depth, LVSI as predictors for recurrence/death (*P* < 0.001) (Table 4).

**Table 4:** Cox survival analysis of patients after radical cervical cancer surgery in HPV-positive and HPV-negative groups.

Factor	*P*-value	HR	95% CI	
			LOWER	UPPER
*Before matching*				
OS				
HPV status	0.013	1.472	1.023	2.118
FIGO staging	0.018	1.021	1.002	1.040
Depth of cervical stromal invasion	< 0.001	1.785	1.423	2.239
LVSI	< 0.001	1.637	1.212	1.983
DFS				
HPV status	< 0.001	1.613	1.250	2.080
Depth of cervical stromal invasion	< 0.001	1.823	1.423	2.239
LVSI	0.011	1.145	1.023	1.283
*After matching*				
OS				
HPV status	0.011	1.198	1.017	1.447
FIGO staging	0.001	1.323	1.180	1.652
Depth of cervical stromal invasion	< 0.001	1.336	1.208	1.472
DFS				
HPV status	0.001	1.274	1.043	1.487
Depth of cervical stromal invasion	0.002	2.059	1.531	2.502

Variables included in multivariate Cox regression models for OS and DFS differed before and after PSM. Before PSM, age and lymph node metastasis were excluded from the OS model, and FIGO stage from the DFS model; after PSM, LVSI was excluded from both models, and FIGO stage from the DFS model. Variables were selected based on multivariate statistical significance and clinical relevance.

#### After 2:1PSM

Given baseline imbalance and sample size disparity, 2:1 propensity score matching was applied, yielding 1674 HPV-positive and 837 HPV-negative patients. Baseline FIGO stage, pathological type and LVSI were well balanced after matching (Table 3).

The HPV-positive group exhibited lower 5-year mortality (4.0% vs. 5.6%) and higher 5-year OS (94.3% vs. 90.4%, *P* < 0.001). It also had fewer recurrence/death events (7.6% vs. 12.8%) and better 5-year DFS (89.2% vs. 82.2%, *P* < 0.001) (Fig. 4).

Multivariate analysis identified HPV status as an independent prognostic factor for OS (*P* = 0.011) and DFS (*P* = 0.001) (Table 4).

After multiple imputation, the prognostic HR and 95% CI of HPV status for OS and DFS remained statistically significant, consistent with the primary complete-case analysis, verifying the robustness of our main conclusions (Table S2).

## Discussion

This multicenter real-world study enrolled a total of 63,926 patients from 47 hospitals of different tiers and types across multiple regions of China. It mainly explored the influence of HPV infection status on the prognostic survival of patients with cervical cancer, and could well reflect the survival profiles of cervical cancer patients with different HPV statuses in the Chinese population. HPV-negative status was verified to be an independent prognostic factor for mortality and recurrence-related survival outcomes in cervical cancer patients.

Previous studies have reported that 4%–52%of cervical cancers are HPV-negative^10^,with considerable heterogeneity in the proportion of HPV-negative cases across different cohorts.A meta-analysis including 243 studies and 30,848 patients with invasive cervical cancer published between 1990 and 2010 demonstrated a downward trend in the prevalence of HPV-negative disease^11^.Specifically,the proportion of HPV-negative cervical cancer was 14.1%during 1990–1999,declined to 12.1%in 2000–2005,and further dropped to 7.1%in 2006–2010.This decreasing trend is largely attributable to continuous improvements in the sensitivity and specificity of HPV detection assays.As recommended in the 2026 Chinese Expert Consensus on Pathological Diagnosis of Non-HPV-Related Cervical Cancer,aided by high-sensitivity HPV testing,the HPV-negative rate of cervical squamous cell carcinoma in the Chinese population is approximately 5%–12%,while a markedly higher rate is observed in cervical adenocarcinoma^12^.The proportion of HPV-negative cervical cancer in the present study was 10.6%,which is consistent with the results of previous investigations.

The molecular mechanisms of HPV-negative cervical cancer are markedly distinct from those of the HPV-positive subtype. The HPV E6 and E7 oncoproteins primarily initiate and promote cervical carcinogenesis by mediating the degradation of p53 and Rb proteins, respectively. Accordingly, TP53 generally remains in the wild-type state in HPV-positive cervical tumors, while HPV-negative cases frequently carry TP53 somatic mutations—a molecular alteration closely linked to malignant progression, aggressive clinical phenotypes, and unfavorable prognosis^13–16^. Nevertheless, the intrinsic molecular regulatory networks driving HPV-negative cervical cancer remain poorly defined, warranting further in-depth investigations to elucidate its underlying pathogenic mechanisms.

The clinical profiles of HPV-negative cervical cancer differ substantially from those of the HPV-positive counterpart. A cohort of 212 cervical cancer patients demonstrated that the median age at diagnosis was 11.9 years younger in HPV-positive cases relative to HPV-negative counterparts (*P* < 0.01)^17^. Another investigation including 760 patients with cervical adenocarcinoma indicated that HPV-positive patients presented at a younger age, whereas rare histological subtypes such as clear cell carcinoma and gastric-type adenocarcinoma were disproportionately enriched in the HPV-negative group.Nicolas et al.^14^ reported that HPV-negative cervical cancer is predominantly of non-squamous histology and tends to be diagnosed at an advanced clinical stage. Patients with HPV-negative disease exhibited a significantly older age at onset, a higher proportion of non-squamous cell carcinoma (*P* < 0.05), and a markedly elevated adenocarcinoma proportion (17.3% vs. 7.4% in HPV-positive cases), which is in line with prior epidemiological evidence. Accumulated evidence further demonstrates that HPV-negative cases are more prone to advanced-stage presentation (*P* < 0.001).Consistently, our study revealed that HPV-negative cervical cancer was mostly diagnosed at late FIGO stages^18^, accompanied by higher rates of lymphovascular space invasion (LVSI) (*P* = 0.001) and deeper cervical stromal invasion (*P* = 0.002). Clinical stage is a well-recognized independent prognostic factor for disease progression and survival outcomes in cervical cancer patients^19^. In the present cohort, 68.7% of HPV-negative tumors were identified at advanced stages, strongly implying an inferior clinical prognosis. Our findings are consistent with earlier reports, supporting that HPV-negative cervical cancer is closely correlated with unfavorable survival outcomes^20,21^ and represents a more aggressive disease subtype.A recent meta-analysis further validated a better prognosis in HPV-positive cervical cancer^22^. In our study, the mortality risk of the HPV-negative group was 1.545-fold higher than that of the HPV-positive group. Accordingly, we conclude that HPV-negative status serves as an adverse prognostic indicator and an independent prognostic risk factor for cervical cancer.The disparities in clinical features and prognostic outcomes between HPV-negative and HPV-positive cervical cancer—including advanced-stage predominance, increased LVSI incidence, and inferior 5-year OS and DFS—were consistent across patients undergoing radical surgery.Notably, this is an observational clinical study. It can only establish an association between HPV-negative status and poor prognosis, rather than confirm a definite causal relationship. Additionally, unadjusted confounding variables such as tumor stage, pathological type, and treatment strategy may also contribute to the survival discrepancy between the two groups.

This study has multiple methodological strengths. Based on a large sample size and multicenter study design, the 2018 FIGO staging criteria were uniformly adopted. Propensity score matching (PSM), center stratification, and sensitivity analysis were performed to effectively control confounding bias, thereby improving the reliability and generalizability of the research findings.

Meanwhile, several limitations of this study remain. First, as a retrospective analysis, the study inevitably has inherent selection bias. Second, a considerable number of clinical data (especially tumor diameter) were missing, which may exert a potential impact on the results of Cox regression and PSM matching. Third, this study covered a 14-year period and involved 47 medical centers. All centers adopted the same HPV detection assay, but subtle differences in experimental procedures and testing conditions existed across institutions during the long study period, and complete standardization could not be achieved. In addition, the actual treatment regimens cannot be presumed to be fully balanced between HPV-positive and HPV-negative groups. Significant between-group differences in FIGO stage and pathological characteristics may directly affect clinical treatment decisions, and residual treatment heterogeneity among centers could not be completely eliminated in this multicenter retrospective study. Although we performed a subgroup analysis focusing on patients receiving surgical treatment, residual heterogeneity regarding surgical approaches and indications for adjuvant therapy still existed across different centers. Moreover, baseline disparities between HPV-positive and HPV-negative patients may influence treatment allocation, so the balanced comparability of treatment regimens between the two groups cannot be fully confirmed. Due to limitations of the original clinical data, detailed information on individual treatment regimens was not fully available, and we were unable to create a statistical table of treatment strategies stratified by HPV status. HPV genotyping data were not available in this study, making it impossible to further analyze prognostic differences among different HPV subtypes. The median follow-up was 49 months for the overall cohort and 59 months for the radical surgery subgroup. We fully accounted for censored cases before the 5-year landmark in survival analysis, which ensures the completeness and reliability of the 5-year survival estimates. Furthermore, FIGO staging tends to act as a mediator variable rather than a simple confounding variable; the HR values after multivariate adjustment may underestimate the true adverse effect of HPV-negative status on the prognosis of cervical cancer.Finally, this retrospective multicenter study lacked systematic recording of immune-related baseline information, including HIV infection status, organ transplantation history. Furthermore, all enrolled patients received treatment before 2018. Given the clinical practice in China at that time, immune checkpoint inhibitors were not routinely used for cervical cancer. None of the cases in this study received immunotherapy, so we were unable to analyze differences in immunotherapy efficacy among patients with different HPV statuses.However, according to the epidemiological features of cervical cancer in the Chinese population, cases with immune dysfunction are extremely rare in routine clinical practice, and the probability of relevant confounding effects on intergroup comparability and prognostic outcomes in our cohort is negligible.

In conclusion, HPV-negative status is an independent adverse prognostic factor for decreased 5-year OS and DFS in patients with cervical cancer, which can significantly affect long-term survival outcomes. Clinically, HPV infection status can be incorporated into the prognostic risk stratification system for cervical cancer patients, providing an important reference for the formulation of individualized follow-up strategies and precise treatment regimens.

## Supplementary Information


Supplementary Information.


## Data Availability

The original contributions presented in the study are included in the article/supplementary material. Further inquiries can be directed to the corresponding authors. Ethics Statement The studies involving human participants were reviewed and approved by Institutional Ethics Review Board of the Nanfang Hospital. Written informed consent for participation was not required for this study in accordance with the national legislation and the institutional requirements.
