# Supplementary material for: The prognostic value of different HPV infection statuses in cervical cancer
Source: Sci Rep. 2026 Jun 14;16:27145. doi: 10.1038/s41598-026-58010-2 (PMC13527069; doi:10.1038/s41598-026-58010-2)
Supplement: Supplementary file 1 [file 41598_2026_58010_MOESM1_ESM.docx]

### Supplementary Table S1. Multivariate Cox regression analysis of HPV status for OS and DFS after multiple imputation.

| Factor | *P*-value | HR | 95% CI | |
| --- | --- | --- | --- | --- |
|  |  |  | LOWER | UPPER |
| **OS** |  |  |  |  |
| HPV status | 0.013 | 1.530 | 1.102 | 2.166 |
| **DFS** |  |  |  |  |
| HPV status | ＜0.001 | 1.540 | 1.224 | 1.977 |

### Supplementary Table S2. Multivariate Cox regression analysis of HPV status for OS and DFS after multiple imputation.

| Factor | *P*-value | HR | 95% CI | |
| --- | --- | --- | --- | --- |
|  |  |  | LOWER | UPPER |
| OS |  |  |  |  |
| HPV status | 0.015 | 1.538 | 1.086 | 2.179 |
| DFS |  |  |  |  |
| HPV status | ＜0.001 | 1.542 | 1.207 | 1.968 |
